# Hashimoto's thyroiditis and hypothyroidism, treated with *ShamanaChikitsa* principles of *Panduroga* – A case report

**DOI:** 10.1016/j.jaim.2025.101172

**Published:** 2025-08-30

**Authors:** P.K.V. Anand, Vaishali Deshpande

**Affiliations:** Department of Kayachikitsa, Parul Institute of Ayurveda and Research, Parul University, Vadodara, Gujarat, 391760, India

**Keywords:** Hypothyroidism, Hashimoto's thyroiditis, Pandurogam, Kanchanara guggulu, Galagandam

## Abstract

Hypothyroidism as a sequel to Hashimoto's thyroiditis is a very common thyroid illness. The conventional treatment is replacement of thyroid hormone to compensate insufficient production from the gland. The clinical features manifested is of widely variable intensity and may not be proportionate always to the blood levels of hormones. The disease does not have a direct mention in Ayurvedic classics. In such instances, treatment principle is adopted from the principles of treatment mentioned for those diseases which holds a similarity in etiopathogenesis, based on the clinical presentations and Dosha-Dooshya analysis. Thus, treatment principles of various diseases such as Galagandam, Agnimandyam, Sthoulyam etc., are being used for the treatment of thyroid diseases. A case of hypothyroidism presented with clinical features similar to Pandurogam was managed with the medicines described in the context of Pandurogam and Sopham. Higher TSH levels were brought to normal limits and high anti thyroid peroxidase level was reduced with a set of Ayurvedic medicines for a longer duration. Keeping away from the popular translation of Pandurogam as Anemia, a symptom oriented diagnostic work up based exclusively on the Ayurvedic classics, followed by symptom-oriented selection of medicines suited to the diagnosis is effective in reducing both symptoms and blood values. This gives the hope of managing hypothyroidism with alternate treatment methods other than supplementation of hormone for lifelong.

## 1 Introduction

Hormones from thyroid gland has a big influence on every cell, tissues, organs and systems in the body. It regulates the metabolism and deals with energy level of the body. Diseases that affect thyroid glands usually presents either a hyper or hypo activity of the gland. They affect the work efficiency and quality of life of individuals and the productivity of society as a whole. Hypothyroidism is the commonest manifestation among them.

As iodine deficiency is a major cause of thyroid illnesses, iodized salts were used in India since 1983 [1]. In spite of 3 decades of iodization program, hypothyroidism in India is estimated to affect one in ten adults. In spite of easier techniques of detection and inexpensive treatments, it has a higher prevalence (10.95 %) [2] in India compared to UK (3.5 %) [3] or USA (4.6 %) [4] or USA and is generally on an increase globally. Women are nearly 8 times more affected by the illness than men [5].

More than a half of the people that suffers from any thyroid diseases are not aware of their illness. A recent study in a costal district of Kerala showed a prevalence of hypothyroidism in the population as 9.6 %. Most of them were diagnosed for the first time during the study exposing the iceberg phenomenon of undiagnosed hypothyroidism existing in the society. 21 % of the participants who were on medication for hypothyroidism showed higher TSH values reflecting the need for sufficient treatment [6].

Another important cause of hypothyroidism is Hashimoto's thyroiditis, which is a disease of autoimmune origin. Its diagnosis is made by clinical manifestations denoting thyroid under activity and positive anti-thyroid antibodies, along with a small goiter enlargement of the thyroid gland. Their elevated TSH levels are attended with thyroxin (T4) supplementation therapy.

There are said to be some influential factors that modulate the response of levothyroxine (L-T4) replacement dose. Many findings imply that thyroid hormone conversion efficiency is an important factor to the biochemical responses to L-T4. Hence levothyroxine dose escalation may have limited success to raise FT3 to appropriate levels in some cases [7].

Researchers has questions on dependency only on TSH level to regulate the dose of L-T4 treatment. The dynamic equilibrium between TSH and thyroid hormones, especially that of free T3 differ in health and diseases. Hence the realization that the TSH level of a healthy normal population cannot be taken to be ideal for a patient on L-T4 medication [8].

Clinical scoring guides can help to detect hypothyroidism at times, when TSH and T4 alone might place people in an expanding grey area of uncertain diagnosis. In 2003, researchers reconfirmed the usefulness of the 1997 clinical score for hypothyroidism, proving that patients' clinical score, ankle reflex response, total cholesterol, and creatine kinase significantly correlated with T4 and T3 levels. In 2011, a review of thyroid clinical scoring scales reaffirmed their continued relevance for diagnosis [9]. The research found that TSH did not strongly correlate with signs of “tissue hypothyroidism” throughout the body. Symptoms shouldn't have to agree with TSH. Research on the pituitary and hypothalamus has revealed TSH is a unique, local, organ-specific response that cannot speak for the rest of the body's T3 sufficiency.

There are a lot of dissatisfied hypothyroid patients looking for ayurvedic management for the disease. Insufficient relief from the clinical manifestations in spite of well-regulated TSH levels with adequate dose of L-T4 may be one reason for this. Apart from this there might be so many other factors like dislike to the lifelong dependency on medication, sporadic manifestation of side effects, hopes vested with Ayurveda to find alternate choices and so on. In autoimmune thyroiditis, the TPO antibodies hardly ever gets normalised even after keeping good hormonal control for long time. As Ayurveda has shown its effectiveness in many autoimmune diseases, there are increasing hopes to find an effective approach through Ayurveda, in thyroid diseases.

### 1.1 Case report

A case of Hashimoto's thyroiditis with hypothyroidism was managed based on the clinical complaints presented and their diagnostic analysis based on Ayurvedic texts with marked results. Ayurvedic treatment protocol, tailored to suit the individual situation in a case of hypothyroidism can be so effective when supported with appropriate correlations and interpretations of classical descriptions.

## 2 Patient information

### 2.1 De-identified patient data

A female aged 63, living in Thrissur, presented in the out patient department of hospital in June 2023, with a recent blood investigation report of high TSH.

### 2.2 Patient concerns & symptoms

She had complaints of fatigue, sleepiness, breathlessness, feeling heavy and cloths gets too tight often with a feeling of swelling all over. She approached hospital OP unit searching for alternate options. She was worried about failing to finish her household chores properly.

### 2.3 Past interventions & outcomes

She was a house wife, apparently well until July 2022, developed neck pain and shoulder pain. Her blood checkup showed hypothyroidism and was put on tab Lethyrox 25mg/day for 3 months. She was also given topical pain relievers, calcium suppliments and Vitamine D supplement. On follow up after 3 months, she was put on Thyronorm 37.5mg/day for 2 months. Anti thyroid peroxidase antibody level was >600 IU/ml, confirming the presence of Hashimoto's thyroiditis. After 2 months the result showed a normal TSH value but persistent symptoms. Generalised swelling, weight gain and fatigue etc., were not relieved while breathlessness and a minimal body pain were also present. She was advised to continue the L.thyroxine at the same dose further. Since 2 or 3 weeks, she felt like the persisting physical complaints were increasing, prompting her to check the TSH value again in May 2023. Her TSH level was found 24.30 IU/ml, in spite of regular dose of Levothyroxine. She was not diabetic, hypertensive or with any other comorbidities and did not have any relevant past history of illnesses.

## 3 Clinical findings

She had drowsy and fatigued facies. Obese appearance with too tight clothing. Appeared dull and slightly indifferent to conversations. Hair was oily and did not complain of hair loss. There was no swellings or nodules visible or palpable in the neck. Skin was dry and with sporadic scratch marks. She was pale looking and with a body language expressing slight irritability. She was complaining of panting if walked a bit fast, feeling of heaviness all over, prefers to remain silent and to sleep whenever she has an opportunity. Occasional palpitations, constipation, a generalised body pain, feeling fullness of stomach and acid belch often, cold extremities were other complaints. She had an uneventful menopause at the age of 48.

She was from costal region, habituated with sea foods. Although currently suffers from fatigue now, her physical strength and digestive capacity were madhyamam until recently. She had many features showing both *Rasadhatu Kshaya* such as *rookshata*, *glani* etc., and *rasavahasrotodushti* such as *asraddha*, *hrillasa*, *gouravam*, *tandra*, *angamarda*, *srotorodha*, *agninasa* etc. Presence of goiter and related manifestations is a feature of *mamsavahasrotodushti*. *Medovahasrotodushti* manifests in her as *sthoulyam*.

The Billewicz diagnostic index [10]. mentioned above was in use at a time when modern methods of diagnosing hypothyroidism were not available. Being easy, inexpensive and reliable, the index is relevant and used even now clinically and for primary screening for prevalent studies etc. [6].

## 4 Timeline

Her Billiwicz score was assessed at her first visit to substantiate the clinical presentation. Please refer to Table 1. The clinical complaints, lab values and the medicine advised are detailed in Table 2.

**Table 1:** Billewicz score.

Symptoms		Present	Absent	2-6-23	4-10-23	9-2-24
1	Diminished sweating	+6	−2	+6	+6	+6
2	Dry skin	+3	−6	+3	+3	+3
3	Cold intolerance	+4	−5	+4	−5	−5
4	Weight increase	+1	−1	+1	+1	−1
5	Constipation	+2	−1	+2	−1	−1
6	Hoarseness	+5	−4	−4	−4	−4
7	Deafness	+2	0	0	0	0
Signs						
8	Slow movements	+11	−3	+11	+11	+11
9	Coarse skin	+7	−7	+7	+7	+7
10	Cold skin	+3	−2	+3	+3	+3
11	Periorbital puffiness	+4	−6	+4	−6	−6
12	Pulse rate	+4	−4	+4	−4	−4
13	Ankle jerk	+15	−6	−6	−6	−6
A score of +25 or more suggests hypothyroidism, while a score of −30 or less excludes the disease				35	5	4

**Table 2:** Clinical visits and complaints.

Date	Complaints	Lab values	Medicines followed
18.7.22	Fatigue & irritability	TSH 12.51	
		FBS 80 mg/dl	
		S.Cholesterol 240 mg/dl	
27.7.22	Shoulder pain & neck pain, fatigue	TSH 14	Lethyrox 25mg/day
			Vitamin D
			Calcium
			Topical pain killer
29.7.22		USG on thyroid reported thyroiditis and a well defined nodule in the isthmus	FNAC showed no malignant cells
27.10.22	Shoulder pain & neck pain reduced.	TSH 8.15	Thyronorm 37.5/day
		Anti thyroid peroxidase antibody >600	
18.1.23	Body ache, fatigue & weight gain	TSH 0.724	
29.5.23	Body ache, fatigue & weight gain, panting	TSH 24.30	
2-6-23	Body ache, fatigue & weight gain, panting		Ayurvedic medicines started. Thyronorm continued
19.7.23	Feeling comfortable	TSH 1.1	Thyronorm stopped
3.10.23	Feeling better	TSH 2.26	Ayurvedic medicines to continue
9-2-24	Feeling normal	Anti thyroid peroxidase antibody 180	
11.3.24	Feels normal	TSH 3.16	

## 5 Diagnostic assessment

### 5.1 Diagnostic methods

The laboratory tests confirmed the presence of auto-immune thyroiditis and consequent hypothyroidism. Nidana Panchakam (five diagnostic principles) were useful in identifying the disease by matching with known Samprapthi of Ayurvedic classics. Dasavidha Pariksha (tenfold examinations) provided sufficient details of the patient which are necessary to generate a treatment plan based on the diagnosis.

Laboratory tests performed in 2022 and the response to the l-Thyroxine prescribed by the general physician proved the diagnosis of Hashimoto's thyroiditis with hypothyroidism. But the upsurge of TSH value while under medication and aggravation of clinical complaints in 2023, she sought Ayurvedic treatment.

### 5.2 Diagnostic challenges

Approach of Ayurvedic practitioners to endocrine diseases is a challenge as it varies widely, based on the concepts considered in the diagnostic and treatment discussion. The commonest concepts and approaches in reported treatments bases on the concept of *dhatwagni mandya*. Correlating goiter with *galagandam*, hypothyroidism is also treated with medicines mentioned in *galaganda chikitsa*, like Kanchanara Guggulu. There are patients undergoing *panchakarma* *chikitsa*, focusing more on *vamana* *karma*, where Vaidya considers hypothyroidism as a *kaphadosha* dominant status [11]. In another case study reported, patient was provisionally diagnosed as *kapha avrita vata* (*vata* obstructed by *kapha*) with *pittaanubandha* (associated with *pitta*) based on the presenting complaints. Here, the treatment protocol containing *rookshana*, *snehana*, *vamana* and *virechana* followed by *shamana* & *rasayana* was also reported to be effective in reducing both TSH and TPO values. There has been case studies of treating hypothyroidism with Yoga & *Pranayama*. There are case studies focusing on Deepana & Pachana following concepts like *agnimandya* leading to *ama* and generation of toxins ending up in the disease [12]. Considering the importance of *rasadhatu*, *kaphadosha*, *jadharagni* and *dhatwagni* in this disease, use of medicines like *arogyavardhini* pills are also reported [[13], [14]]. Treatment approaches considering the clinical manifestation and individualised management is also often reported. *Sthoulyachikitsa* has also been considered as a treatment approach in some reports.

Such wide variations in diagnosis and treatment plan for hypothyroidism is due to wide variations in the clinical presentations in them. The diagnostic approach of Ayurveda prefers to identify the individual and his illness in a larger comprehensive picture, rather than a deficiency of a constituent substance like thyroid hormone. Supplementing the deficient substance is correction of *dhatukshaya*, hence a part of treatment. Along with that, correction of causes for *dhatukshaya* and the management of symptoms and complications are equally important. Thus the clinical presentation of the individual was analysed and matched with known *samprapti* of diseases described.

### 5.3 Diagnosis

Hypothyroidism is an *Anuktavyadhi* (a disease manifestations not mentioned in Ayurvedic texts) [15]. The clinical picture patient presented was very similar to the description of *panduroga* description by Vagbhata [16]. She presented 13 features out of 18 general features described in the context. She also had 6 prodromal features out of 8 poorvaroopa features of panduroga. She presented body ache, swelled up body, loss of taste and dry feel in mouth, blotted abdomen and dry and constipated stools from the list of *vata* dominant *panduroga*. Occasional *amlaka* (acid belch) and *tandra* (drowsiness) from the list of features of *pitta* and *kapha* dominant panduroga features respectively. Please refer to Table 3.

**Table 3:** Comparison of features of Pandurogam, Hypothyroidism and those presented by the patient.

	Features of Panduroga	Features in hypothyroidism	Complaints of the patient
1	*Gouravam*	Weight gain/Feeling heavy	Dress getting tight, feeling heavy, weight gain
2	*Dhatu Saidhilyam*	Reduced metabolism	Assumed by presence of hypothyroidism
3	*Oja Gunakshayam*	Reduced energy	Loss of initiation to activity
4	*Alpa rakta*	Not seen	Not seen
5	*Alpa Medas*	Hyper cholesterolemia	Medas getting stagnant in Rakta
6	*Nissara*	Not assessable	Not assessable
7	*Sladhendriya*	Visual disturbances, hearing impairement, cold touch, constipation, reduced libido, hoarseness of voice, sluggish movements	Cold touch, constipation and slow movements
8	*Anga marda*	Generalised body ache	Body ache
9	*Hridravam*	Palpitations	Palpitations
10	*Soonakshikootah*	Peri orbital edima	Edima present
11	*Sadanah*	Fatigue	Fatigue
12	*Kopanah*	Irritability	Mild irritability
13	*Shteevanah*	Not seen	Not seen
14	*Alpavak*	Less talkative	Only answering to questions in few words
15	*Annadvit*	Not seen	Not seen
16	*Sisiradweshi*	Cold intolerance	Cold intolerance
17	*Seernaromah*	Hair fall	Not seen
18	*Hata analah*	Reduced metabolism	Assumed by the presence of hypothyroidism
Poorvaroopas of *Panduroga*			
1	*Hridaya spandanam*	Palpitations	Palpitations
2	*Twachi rookshata*	Dry & course skin	Dry & course skin
3	*Aruchi*	Not seen	Not seen
4	*Peeta mootratwam*	Not seen	Not seen
5	*Sweda abhavah*	Reduced sweating	Reduced sweating
6	*Alpa vahnita*	Not seen	Not seen
7	*Sadah*	Tiredness	Tiredness
8	*Sramah*	Breathlessness on activity	Breathlessness on activity

#### 5.3.1 Analysis of symptoms (Samprati-Lakshana Sambandha)

Generalised swelling was the clinical picture the patient presented. This has contributed to the increase in her body weight and also marked subjective feeling of heaviness (*Gouravam*). Flabby appearance of the body (*Dhatu saidhilya* – as seen by asamhata Sareera), reduced properties of Ojus (as seen by insecure feelings – *Bhibheti*).

The conventional and popular translation of the disease Panduroga is anemia. Reduced quantity of Rakta is a feature of Panduroga, which is easily correlated with anemic status. Arunadatta, the commentator says that ‘Raktadhatu’, the refined final produce after the Dhatu Parinama sequence is not generated in Panduroga, instead it stays in the form of Pitta, the waste product [23]. As the patient did not present a deficient haemoglobin status, we assume the kind of *pitta* involvment in this patient is not in the form of anaemia; instead, fluidity (*drava guna* of *pittadosha*) is increased here as seen by the generalised edema.

Reduced quantity of *medas* is also a feature of *panduroga*. The stagnation of *dhatu parinama* sequence is obvious here by the association of hypercholesterolemia with hypothyroidism. Conversion and metabolism of lipids are sluggish since the *rasa-rakta* *dhatus* hold the under-utilised, stagnant, higher than normal levels of lipoproteins i.e. derivatives of *medo* *dhatu* in blood. Sweat is the waste generated during the conversion of *medodhatu.* Reduced sweating also shows reduced Dhatu parinama by *medodhatwagni*. Keeping the *dhatusara* to the optimal levels will be hampered by reduced Dhatuparinama leading to substantial loss vigour & vitality (Nissarah).

Sladhendriya, another feature of *panduroga* means the 5 *jnanendriyas*, 5 *karmendriyas* and mind are not working in the optimal level. In hypothyroidism, these features are seen in variable intensities manifested as visual disturbances, hearing impairement, cold touch, constipation, reduced libido, hoarseness of voice, sluggish movements etc.

Angamardam or generalised body ache is a *vata* dominant feature which is frequently seen in *rasadhatu* related pathologies such as menstrual abnormalities, *jwara* etc. *Hridravam* shows the involvement of *moolasthana* of *rasadhadhatu* i.e., *hridayam* and is manifested as palpitations.

Hypothyroidism may cause reduced secretions in stomach and dry mouth due to lesser salivation, leading to reduced taste sensation. *Shteevanam* (salivation & spitting), *hata analah* (reduced digestion), *aruchi* (reduced taste in foods) which are among the general and prodromal features list of Pandurogam, are not among the clinical features of hypothyroidism and not presented in the patient also. Acid belch is not so uncommon in patients taking Levothyroxine regularly. Amlaka is a feature of *pittadosha* in *Panduroga*. Thyroiditis, which is the cause of hypothyroidism, itself is an inflammatory pathology, showing a persistant and progressive *pachyamana* status dominated by *pitta dosha*. Hence *pittadosha* *shamana* by *virechana* is important here.

Iodine deficiency due to cell damage in autoimmune thyroiditis, causing hypothyroidism shows the reduced nutrient function of *rasadhatu*. An essential nutrient like iodine is not sufficiently available at the site and the thyroid secretions are not supplied all over the body due to insufficient production. This leads to *dhatukshaya* in subsequent *dhatus* like *rakta*, *mamsa* etc., triggering *vatakopam* in those *dhatus*. Due to reduced metabolic rate and overall activities *Vatakshaya* happens along with *kaphasanchayam* happening due to the lack of conversion at *rasadhatu*.

Accumulation of *asthayi* *rasadhatu* shows *rasadhatu vikaras* due to *rasadhatuvridhi*. Hence the clinical features of hypothyroidism has features of Rasadushti. Thyroid secretions are related with metabolic functions, which is translated as *agni* related functions of the body. Since both *rasadhatu* and *raktadhatu* are the seat of *pittadosha*, which has the function of *pachanam*, and they both travel together in the body with functions of nourishment and metabolism.

When the clinical presentation is with *sopham*, which is a complication of *pandurogam*, the *samprapthi* is more related to *raktakshayam* and resultant *rasavridhi* due to increase of *asthayi* *rasadhatu* in the *rasa-rakta* complex. Hence, the clinical manifestations of *rasavridhi*, in hypothyroidism may be understood as an event during the course of *panduroga* *samprapti.* When s*opham* (inflammation/inflammatory swelling) is presented, it can be taken as panduroga Samprapti has entered into next stage during its course where its *upadravas* like *sopham* has set in. At this stage of Samprapti, instead of *rasapradoshaja* treatments, *pandu-sophahara* treatments are to be followed. The treatment plan of *Panduroga* is *snehanam*, *vamanam*, *srothasodhanam* and *Shamanam*, which clears blockage of channels due to accumulation of *rasadhatu* and increases the constituents of *raktadhatu*.

#### 5.3.2 Prognosis

Alternating between hyperthyroidism and hypothyroidism phases before settling finally as hypothyroidism is commonly found in Hashimoto's thyroiditis. Unlike this, after a period of good control on laboratory values with biomedical intervention, worsening clinical complaints and increased TSH prompted her to seek Ayurvedic treatment. *Sleshma*, *twak*, *rakta* & *mamsa* are the substances getting affected in the pathogenesis of *panduroga*. *Vagbhata* says in *panduroga*, Vaidya should also apply the treatments mentioned in *sopham* [24]. The features of *pandu* & *sopham* are curable here, but the underlying hypothyroidism due to irreversible damage to the organ makes the condition *yapyam*.

## 6 Therapeutic interventions

### 6.1 Type of therapeutic intervention

A *Shamana Chikitsa* Plan of care was prepared for initial management, with a longer duration of medication in focus. *Lekhana*, *pachana*, *mrudu* *virechana* with formulations relevant to the diagnosis were selected based on their *Rasayana* and *prasadana* potentials.

### 6.2 Internal medications

The prescription given on 2-6-23 was as follows. (see Table 4)

**Table 4:** Initial prescription.

Sl.No.	Oushadham	Maatra	Anupanam	Frequency	Oushadhakalam
1	*Vyoshadi kashayam* [17]	15 ml	Warm water	Twice a day at 6 a.m. and 6 p.m.	In empty stomach
2	*Mandoora Vatakam* [18]	1 tablet	Warm water	Twice a day	After food
3	*Avipathy choornam* [19]	1 teaspoon	Warm water	Once a day	Before supper
4	*Kanchanara Guggulu* [20]	2 tablets	Warm water	Twice a day	After food
5	*Dasamoola hareethaki* [21]	1 big spoon	Nil	Once a day	After supper
6	*Chandraprabha* tablets [22]	2 tablets	Warm water	Twice a day	Before food

### 6.3 Changes in therapeutic intervention

On 26-6-23 patient reported occasional mild stomach pain. In addition to the current medications, she was prescribed *dadimashtaka choornam* [25] −1 tsp with warm water as and when the pain appears. On 3-7-23 she reported good and quick relief from stomach pain when *dadimashtaka choornam* is taken. On 10-7-23 the patient reported frequent stomach pain and tenesmus in mild intensity. Using *avipathy* *choornam* to ensure laxation was stopped and advised to use only as and when required. Dadimashtaka choornam was made a regular daily medicine twice daily before food. This event may be interpreted as a sign of *rookshata* developed in the *koshta* as a result of medications.

## 7 Follow-up and outcomes

The results of intervention were recorded using score sheets before treatment, after medication for 4 months and after medication for 8 months. Refer to Table 5, Table 6.

**Table 5:** Zulewski's clinical score for hypothyroidism.

On the basis of symptoms		Present/Absent	2-6-23	4-10-23	9-2-24	
1	Diminished sweating	Sweating in a warm room	1/0	1	1	1
2	Hoarseness	Speaking voice/singing voice	1/0	0	0	0
3	Parasthesia	Subjective sensation	1/0	0	0	0
4	Dry skin	Dryness of skin noticed spontaneously	1/0	1	1	1
5	Constipation	Bowel habit, use of laxatives	1/0	1	0	0
6	Impairment of hearing	Progressive impairment of hearing	1/0	0	0	0
7	Weight increase	Recorded weight increase/tightness of cloaths	1/0	1	0	0
Physical signs						
1	Slow movements	Observe patient movements	1/0	1	1	1
2	Delayed ankle reflex	Observe the relaxation of the reflex	1/0	0	0	0
3	Coarse skin	Examine hands, forearms, elbow, for roughness and thickening of the skin	1/0	1	1	1
4	Periorbital puffiness	This should obscure the curve of the malar bone	1/0	1	0	0
5	Cold skin	Compare temperature of hand with examiners	1/0	1	1	1
Sum of all signs present	12/0	8	5	5		

Zulewski et al. set out to re-evaluate the classical signs and symptoms of hypothyroidism in the light of modern laboratory tests. A score >5 points defined hypothyroidism, while a score of 0–2 points defined euthyroid. The score is important for the concept of tissue hypothyroidism, given an easy method of assessing its severity. It can be used to evaluate patients with discordant laboratory results, and to monitor effects of therapy [26].

**Table 6:** Clinical improvement score.

	Symptoms	Criteria	Score	2-6-23	4-10-23	9-2-24
1	Puffiness	Absent	0			0
		occasional	1		1	
		Periorbital edima/puffiness in the morning every day, relieved in later part of the day	2	2		
		Persistent	3			
2	Edema	Absent	0			0
		Edema over lower/upper extremities	1		1	
		Edema over both extremities	2			
		Edema all over the body	3	3		
3	Dry and coarse skin	No dryness	0			
		Dryness after bath only	1	1	1	1
		Dryness relieved by oil application	2			
		Dryness not relieved by oil application	3			
4	Breathlessness	Absent	0			
		Occasionally, after strainous workout	1		1	1
		Even climbing stairs, relieved by rest	2			
		Felt in routine works like bathing, changing cloths etc	3	3		
5	Constipation - Frequency	Once a day	0		0	0
		Once in two days	1	1		
		Once in 3 days	2			
		Once in more than 3 days	3			
	Constipation – Consistancy	Sidhila	0			
		Madhyama	1		1	1
		Kathina	2	2		
		Grandhila	3			
	Constipation – Straining	No	0		0	0
		Occasionally/Bearable	1	1		
		Frequently/Severe	2			
6	Weakness	Able to do exercise without difficulty	0			0
		Able to do mild excercise	1		1	
		Able to do only mild work	2			
		Able to do mild work with difficulty	3	3		
		Not able to do even mild work	4			
		Not able to do even day-to-day routine works	5			
7	Lethargy	Finishes work satisfactorily with good vigour and in time	0		0	0
		Without desire/unsatisfactorily but in time	1			
		Without desire/unsatisfactorily/with lot of mental pressure and not in time	3	3		
		Doesnot have any initiation and not want to work even after pressure	4			
8	Fatigue	Normal	0		0	0
		Prefers standing than walking	1			
		Prefers sitting than standing	2			
		Prefers lying than sitting	3			
		Prefers sleeping than lying down	4	4		
9	Generalised Muscle ache	No ache	0		0	0
		Relieved by rest	1			
		Not relieved by rest but by external application	2			
		Not relieved by extenal application alone but by internal medication	3	3		
		Present always in spite of medications	4			
10	Duration of Menstual bleed	4–7days	0	NA	NA	NA
		3 days	1			
		2 days	2			
		1 day	3			
11	Intervel between two cycles	25–29 days	0	NA	NA	NA
		35–39 days	1			
		40–45 days	2			
		>45 days	3			
12	Hair fall	Absent	0	0	0	0
		On washing	1			
		Combing	2			
		Simple stretching	3			
13	Hoarseness of voice	Absent	0	0	0	0
		Present	1			
14	Cold intolerance	Absent	0			
		Present	1	1	0	0
	Sum of all signs present			27	5	3

Even though the earlier 2 scales, namely Billewicz diagnostic index and Zulewski's clinical score for hypothyroidism are widely used and much authentic, it does not address the common complaints in an Indian setting. This scoring [27] is a combination of 2 mutually similar score sheets, which includes the complaints applicable to females only. It also addresses features like hair fall, lethargy, gasping etc., which are commonly seen.

### 7.1 Important follow-up diagnostic and other test results

Even though fluctuations in the TSH values are possible in Hashimoto's thyroiditis, here the TSH values went higher to the level of 24.3 on 29.5.23 in spite of its initial success at the same dose as seen from the results TSH 0.724 on 18.1.23 which was earlier TSH 8.15 on 27.10.22. Within 4 days after the higher value, Ayurvedic medicines on along with the Levothyroxine in same dose was started. In six weeks the TSH value came down to normalcy on 19.7.23 to the level of 1.1 along with significant relief in the clinical complaints. This could be due to better bio-availability offered by the addition of Ayurvedic medicines to the prescription.

Levothyroxine was stopped as the TSH level found normal on 19.7.23 and only Ayurvedic prescription was continued. After 6 weeks on 3.10.23, the TSH level was at 2.26 showing normalcy. This indicates the Ayurvedic prescription alone, with no thyroxin supplements along with was able to keep TSH level in normalcy. At 11.3.24 the TSH levels were 3.16, showing normalcy and no noticeable presence of clinical features during this period.

The TPO levels which was more that 600 on 27.10.22 was reduced to 180 on 9.2.24 which shows the inflammatory activity due to Hashimoto's thyroiditis has also reduced. The systemic anti -inflammatory effect that could reduce generalised swelling and other symptoms in this patient could have helped to create a milieu interior that does not provoke auto immune activities in the body as seen by good response anti thyroid peroxidase levels in the serum. In the past two decades, Ayurveda has become popular choice of treatment in various autoimmune diseases such as Rheumatoid arthritis etc.

### 7.2 Intervention adherence and tolerability

The prescription was finalized after discussing the plan with the patient and taking her opinions regarding feasibility and compliance. The prescription was continued with regular weekly follow ups. This ensured intervention adherence and tolerability.

### 7.3 Adverse and unanticipated events

On 26-6-23 patient reported occasional mild stomach pain. In addition to the current medications, she was prescribed *Dadimashtaka choornam*. It is described in the context of *Kaphaja atisara chikitsa*. It is also a widely used choice for stomach pain due to various causes like dysentery, flatulence etc.

## 8 Discussion

### 8.1 Strengths and limitations

This case report demonstrates the need of matching clinical complaints to identify a known *samprapti* so that the clinician can adopt its treatment principles, medicines, *pathya* etc. Positive outcomes can be achieved when the treatment is attended on a case to case basis, in contrast to generalised supplementation irrespective of clinical presentation.

### 8.2 Discussion on relevant medical literature

In this patient, the treatment principles of *pandu* and *sopham* were brought in due to the similarity of clinical complaints with the textual descriptions of *pandurogam* and *sopham*. The selection of medicines from the context is largely based on empirical knowledge and relevance to the purpose of treatment in this case. A comprehensive prescription is generated after considering the opinions of the patient regarding compliance.

Primary indication of *vyoshadi kashayam* is *pandu*. From the ingredients and also from the beneficial outcome, it can be assumed that, it was working at the level of agni, with far reaching benefits at the level of *dhatwagni* and *bhootagni*. *Mandoora* *vatakam* is indicated in *pandurogam*, which is also indicated in *ajarakam* (reduced digestion) and *sopham*. It contains cow's urine, which is among those listed to be given [chapter 17, verse 17–19] in *srotovibandham* (obvious here by reduced sweating and constipation), *agnimandyam* (assumed from the reduced *dhatwagni*) and sluggish stomach (*stimitasayah* - assumed here by constipation and reduced digestion).

*Avipathy choornam* is a widely used safe laxative. It is indicated in *Jwaram*, *Pandurogam*, weak digestive power and in toxin related manifestations. It is considered ideal in *Pitta* diseases. *Pandurogam* is a *pitta* dominant disease. Laxation is an important treatment in Pitta diseases.

*Kanchanara Guggulu* is widely used medicine for *galagandam*. It has been a main object of various clinical studies in hypothyroidism in the past 2-3 decades. *Dasamoola hareethaki* is a popular choice for long term use in generalised edema. It is a broad-spectrum medicine which is also a mild laxative. *Chandraprabha* tablets, which is widely used in urinary system diseases was given here considering its diuretic potential in generalised swelling. The dominant ingredients of these 3 medicines also have *Rasayana* properties.

The Dosha status related manifestations reveal that the patient has presented a *vata* & *kapha* dominant status. When matched with the *Nidana Panchaka*, the features were similar to the disease Pandu and *sopham*. The hetu (causative factor) here is a systemic inflammatory activity (*pakam* – an act of *pitta* *dosha*) of autoimmune origin which had caused damages and degeneration (an act of Vata dosha) of thyroid gland, hampering its function in metabolism. As a sequel, stagnation happens at Rasadhatu, which is presented as increased features of *asthayi* *rasadhatu*, which are similar to the features of increased *kaphadosha*. The *poorvaroopa* and *roopa* are very much similar to those of *pandurogam*. Here the disease *pandu* has presented its important *upadravam* i.e. *sopham* also, which helped to confirm the diagnosis. Using the principles of *panduroga* *chikitsa* and *sophachikitsa*, selecting medicines from that context based on its suitability to the patient's clinical condition and willingness to follow the prescription for sufficient duration has become effective in reducing the sign and symptoms along with laboratory values. This shows the *upsayam* to the treatment along the lines of *panduroga* *chiktsa*.

The medications were effective to make *Srotosodhanam* (removing the stagnations in metabolic pathways) by reducing the vitiated *Kapham*, pacifying the *Pittam* and making *Anulomana* to the *Vatadosha*. The medicines in general worked as *deepana,* *pachana* and *rookshanam* in the situation to make sufficient *Agnideepti* at *Dhatwagni* level, and to make effective *rookshanam* by offering dryness in order to reduce the fluid retention at sites which referred in the texts as location of sopham i.e., between *twak* & *mamsam*.

Patients suffering from Hypothyroidism frequently approach Vaidyas for management of their refractory clinical complaints, alternate options for hormone supplementation and also in search of permanent cure for the illness. Many of such cases treated with Ayurveda are published with reduction in TSH values and improvement of clinical symptoms. Often a few cases of hypothyroidism with increased anti-thyroid peroxidase were also reported with obvious improvement after treatment. Seetha C et al., has reported the effectiveness of a treatment protocol containing *rookshana,* *snehana,* *vamana* and *virechana* followed by *Shamana* drug *Varunadi kwatha bhavita shilajatu* is followed in this case of HT. This protocol is found to be effective in clinical, biochemical and sonological aspects. In this case, reduction of TSH from 46 to 16 IU/ml and TPO from 208.7 to 32 IU/ml was reported before and after treatment. The same treatment protocol has been reported further as a case series of 5 cases with conspicuous reduction in TSH, TPO and Anti TG values in all. Seethadevi et al. has reported TPO value more than 1300 reduced to 299.5 IU/ml in a case of Hashimoto's thyroiditis, with *Shodhana* followed by *shamana* *chikitsa*.

This case presents improvements in clinical features, TSH and TPO values. The clinical presentation of the patient was unique being *sopha* dominant, where the *samprapti* of *pandusopha* is considered for correlation. Thus, treatment line similar to that of *pandu* and *sopha* was adopted here, along with medicines suggested in the context. The case study shows the effectiveness of *Shamana Chikitsa*, with no preceding *Sodhana Chikitsa* in autoimmune hypothyroidism.

### 8.3 Conclusion

Apart from a hormonal supplementation, management of hypothyroidism using Ayurvedic principles and practice involves a highly patient oriented medicine selection based on the clinical presentation and diagnostic analysis explained in Ayurvedic classics.

## 9 Patient's perspective

Patient responded to the consultations very positively and vowed full cooperation throughout the treatment. She was satisfied with the results obtained by the treatment and plans to continue it further for a longer duration.

## Informed consent

Authors certify that they have obtained informed consent from the patient, where they have given their consent for reporting the case along with relevant clinical information in the journal, with due efforts to conceal identity.

## Funding sources

None

## Conflict of interest

The authors declare that they have no known competing financial interests or personal relationships that could have appeared to influence the work reported in this paper.
